# Multiple-factor analysis of the first radioactive iodine therapy in post-operative patients with differentiated thyroid cancer for achieving a disease-free status

**DOI:** 10.1038/srep34915

**Published:** 2016-10-10

**Authors:** Na Liu, Zhaowei Meng, Qiang Jia, Jian Tan, Guizhi Zhang, Wei Zheng, Renfei Wang, Xue Li, Tianpeng Hu, Arun Upadhyaya, Pingping Zhou, Sen Wang

**Affiliations:** 1 Department of Nuclear Medicine, Tianjin Medical University General Hospital, Tianjin, P. R. China

## Abstract

^131^I treatment is an important management method for patients with differentiated thyroid cancer (DTC). Unsuccessful ^131^I ablation drastically affects the prognosis of the patients. This study aimed to analyze potential predictive factors influencing the achievement of a disease-free status following the first ^131^I therapy. This retrospective review included 315 DTC patients, and multiple factors were analyzed. Tumor size, pathological tumor stage, lymph node (LN) metastasis, distant metastasis, American Thyroid Association recommended risks, pre-ablation thyroglobulin (Tg), and thyroid stimulating hormone (TSH) displayed significant differences between unsuccessful and successful group. Cutoff values of Tg and TSH to predict a successful outcome were 3.525 ng/mL and 99.700 uIU/ml by receiver operating characteristic curves analysis. Binary logistic regression analysis showed that tumor stage T3 or T4, LN metastasis to N1b station, intermediate and high risks, pre-ablation Tg ≥ 3.525 ng/ml and TSH <99.700 μIU/mL were significantly associated with unsuccessful outcomes. Logistic regression equation for achieving a disease-free status could be rendered as: y (successful treatment) = −0.270–0.503 X_1_ (LN metastasis) −0.236 X_2_ (Tg) + 0.015 X_3_ (TSH). This study demonstrated LN metastasis, pre-ablation Tg and TSH were the most powerful predictors for achieving a disease-free status by the first ^131^I therapy.

In recent years, the incidence of thyroid cancer is rapidly increasing, and about 90% of which is differentiated thyroid cancer (DTC), including papillary thyroid cancer (PTC) and follicular thyroid cancer (FTC)^1^. For the optimal management of DTC, total thyroidectomy, post-operative radioactive iodine (^131^I) therapy and thyroid stimulating hormone (TSH) suppression treatment are the three most essential methods^2^,^3^,^4^,^5^. Theoretically, ^131^I therapy is thought to reduce the recurrence and mortality rate in DTC patients by destroying microscopic residual tumor foci, which also facilitates early detection of recurrence by enhancing the sensitivity and reliability of serum thyroglobulin (Tg) and ^131^I whole-body scan (WBS)^5^,^6^,^7^. However, there is a lack of consensus among guidelines about the optimal ^131^I dose for ablation^3^,^4^,^5^, although the most recent meta-analysis favored the empiric 100 mCi^6^,^7^.

Although ^131^I therapy is generally considered safe, some potential complications could happen. For instance, a large dose of ^131^I could increase the risk of dysfunction of several body organs, including gastrointestinal, pulmonary, and hematopoietic systems, salivary glands, and gonads^4^,^5^,^8^. Hakala *et al*.^9^ even reported that for young patients (<40 years), the risk of secondary malignancies would dramatically increase. More importantly, the use of ^131^I for remnant ablation to reduce the risk of recurrence following thyroidectomy in low risk DTC patients remained controversial^10^,^11^. On the other hand, recent evidence showed that for low risk DTC patients, low dose of ^131^I therapy could successfully achieve complete ablation^12^,^13^. Therefore, it is imperative that clinicians should utilize individualized and optimized ^131^I dose for the management of DTC patients to balance the risks and benefits of this important therapy.

Predictors of DTC prognosis have been investigated by several studies, although the values of different parameters are inconsistent among them. For instance, pre-ablation stimulated Tg is known to be a good predictor of successful ablation in DTC patients^14^. Pre-ablation Tg has also been demonstrated as an important indicator for predicting persistence or recurrence during follow-up^15^,^16^,^17^. However, cut-off values of Tg for successful ablation or recurrence prediction were quite different among the above mentioned studies. A second good example is about TSH. Guidelines recommended that DTC patients receiving ^131^I therapy should have a serum TSH level of higher than 30 μIU/mL^2^,^3^,^4^,^5^. However, Vrachimis *et al*.^18^ demonstrated that endogenous TSH levels at the time of ^131^I ablation did not influence ablation success, recurrence-free survival or differentiated thyroid cancer-related mortality. Hasbek *et al*.^19^ also found that a high TSH level alone was not a factor for the success of ablation. So, a comprehensive research to assess possible predictive factors would be valuable to the subject.

The goal of this study is to analyze potential predictive values of various clinical factors to achieve a disease-free status by the first ^131^I therapy in post-operative DTC patients.

## Results

### Data comparisons of DTC patients with or without disease-free status

A total of 315 DTC patients were recruited in our analysis. Among them, 118 patients (37.5%) achieved disease-free status, while 197 patients (62.5%) did not. Patient’s age ranged from 18 to 77 years old (47.5 ± 12.2 years). Clinical characteristics of the 315 patients were summarized in Table 1. Between the unsuccessful and successful ablation outcome groups, age and gender had no statistical differences. Most of the patients were PTC, accounting for 100.00% of the successful group, and 97.46% of the unsuccessful group. Pathology showed no statistical significance. Capsular invasion, American Joint Committee on Cancer (AJCC) tumor-node-metastasis (TNM) staging system, time interval between surgery and ^131^ I therapy also had no differences. Tumor size (diameter >1 cm versus ≤1 cm) (X^2^ = 4.668, P = 0.031), pathological tumor stage (X^2^ = 13.193, P = 0.004), lymph node (LN) metastasis (X^2^ = 18.192, P < 0.001), distant metastasis (Fisher exact test, P = 0.001), American Thyroid Association (ATA) recommended risks (X^2^ = 17.453, P < 0.001), pre-ablation Tg (t’ = 6.900, P < 0.001) and TSH (t’ = −3.367, P = 0.001) displayed significant differences.

### Diagnostic values of Tg and TSH for a successful therapy

**Figure 1:**
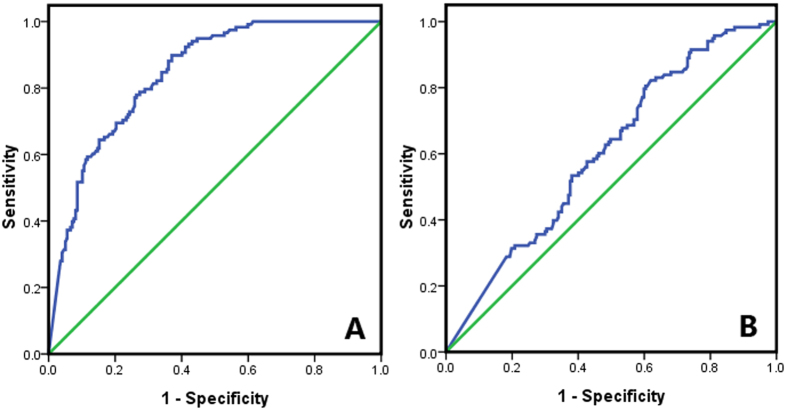
Diagnostic values of pre-ablation thyroglobulin **(A)** and thyroid stimulating hormone **(B)** analyzed by receiver operating characteristic curves.

For continuous parameters (pre-ablation Tg and TSH), receiver operating characteristic (ROC) curves were conducted (Fig. 1). A cutoff value of 3.525 ng/ml for pre-ablation Tg could be used to differentiate successful and unsuccessful ablation with a sensitivity of 77.12%, specificity of 74.12%, PPV (positive predictive value) of 64.09%, and negative predictive value (NPV) of 84.39%. A cutoff value of 99.700 μIU/ml for TSH could distinguish successful and unsuccessful ablation with a sensitivity of 57.63%, specificity of 57.46%, PPV of 44.74% and NPV of 69.33% (Table 2).

### Risk assessments for a therapeutic failure

Next, we used a binary logistic regression analysis to identify risk factors associated with therapeutic failure. No risks were found among age, gender, capsular invasion, TNM stage and time interval between surgery and ^131^I. In contrast, tumor stage T3 or T4, LN metastasis to lateral cervical region (N1b) station, intermediate and high risks, pre-ablation Tg ≥ 3.525 ng/ml and TSH < 99.700 μIU/mL were significantly associated with a therapeutic failure (Table 3).

### Equation for achieving a disease-free status

Using an unconditioned logistic regression analysis, LN metastasis, pre-ablation Tg level and pre-ablation TSH level showed significant associations with the disease-free status and were included in the regression model. The equation for achieving a disease-free status by the first ^131^I therapy could be rendered as: y (successful therapeutic outcome) = −0.270–0.503 X_1_ (LN metastasis) −0.236 X_2_ (pre-ablation Tg level) +0.015 X_3_ (pre-ablation TSH level).

## Discussion

Although a number of studies investigated the predictors of DTC prognosis, inconsistency existed about values of various predictors^14^,^15^,^16^,^17^,^18^,^19^,^20^. The merit of the current study is that it examined all the predictors within a comprehensive statistical framework. This retrospective study demonstrated that LN metastasis, pre-ablation Tg and TSH were the most powerful predictive factors for a successful ^131^I ablative outcome. Existence of LN metastasis, high pre-ablation Tg level and low pre-ablation TSH level in patients with DTC were associated with increased risk for therapeutic failure.

The incidence of PTC is usually much higher than FTC in clinic. PTC is prone to LN metastasis at an early stage with no definite relationship with primary lesion size. Even when the primary tumor is ≤1 cm, LN metastasis could still happen often in PTC^21^,^22^. Most scholars believe that the first station of LN metastases is the central region (N1a), and then the second station to the N1b. However, lateral LN metastasis without central LN involvement could also occur in patients with PTC^23^. Several studies showed that for patients (especially age ≥45 years), LN metastasis was an indicative factor for high recurrence rate and low survival rate. Compared with N1a station, the 5 year survival rate was significantly lower in N1b station^24^,^25^,^26^. Moreover, previous studies^20^,^27^,^28^, including one case report from our institution^22^, found that extensive metastases would affect the overall therapeutic effectiveness of ^131^I. In our study, after multiple-factor analysis, we found that compared with DTC patients without LN metastasis (N0), no significant difference of disease-free status rate was achieved in the N1a patients [odds ratio (OR) = 1.406, P = 0.185]. However, significantly higher risk of unsuccessful therapeutic outcome was found in N1b patients (OR = 4.783, P < 0.001). In the logistic regression analysis, LN metastasis had a coefficient of −0.503, which indicated positive LN metastasis would increase the risk of therapeutic failure. Moreover, in our study, 14 DTC patients had distant metastases, none of them achieved a disease-free status by the first ^131^I therapy.

The clinical value of Tg has been reported in many studies, especially with regard to DTC disease progression or recurrence^14^,^15^,^16^,^17^,^29^. Some studies have shown the relationship between pre-ablation Tg and successful ablation. For example, Lim *et al*.^29^ analyzed various predictors for successful ablation and disease-free status using univariate and multivariate analyses, and determined that a Tg value greater than 5 ng/mL was the most powerful predictor for ablation failure. A meta-analysis including 3947 patients demonstrated that the pre-ablation Tg was a useful negative predictor for persistent and recurrent DTC, in particular NPV was 94% if pre-ablation Tg level was less than 10 ng/mL^17^. Gonzalez *et al*.^15^ conducted an investigation on 133 DTC patients and revealed that a pre-ablation Tg of less than 8.55 ng/mL could predict remission of disease in 18 to 24 months after ^131^I therapy with a sensitivity of 88%, specificity of 72%, PPV of 47% and NPV of 95%, and area under the ROC curve of 0.872. In our study, a baseline-stimulated Tg value of 3.525 ng/mL was identified as the optimum cut-off by the ROC analysis. If Tg was lower than 3.525 ng/mL, the disease-free status by the first ^131^I therapy could be predicted with an accuracy of 75.24%. In the logistic regression equation, Tg had a coefficient of −0.236, which indicates the low pre-ablation Tg level could predict the possible disease-free status. Hence, measurement the pre-ablation Tg in patients with DTC is important.

We also found that pre-ablation TSH level was an important factor for complete remission by the first ^131^I therapy. ATA and European guidelines recommended a dogma that DTC patients receiving ^131^I therapy should have a serum TSH level of higher than 30 μIU/mL^2^,^3^,^4^,^5^. Historically, it was first reported in 1977 by Edmonds *et al*.^30^ that DTC patients who failed to produce a TSH level of >30 μIU/mL by a postoperative levo-thyroxine (LT4) withdrawal preparation showed much lower rates of successful ^131^I ablation. However, several recent studies showed that TSH stimulation for postoperative ^131^I ablation was not important and did not influence the ablation success rate^18^,^19^. In our study protocol, all patients were ideally required to have a TSH level up to 30 μIU/mL before treatment, yet there were 19 patients who didn’t meet this requirement. We gave them 100 mCi of ^131^I, nonetheless. As a result, in these 19 patients, only 2 achieved a disease-free status after the first ^131^I therapy. In the 17 patients who were not successfully ablated, 2 had distant metastases, and 4 were in N1b stage. This result indicates that remaining functional benign and malignant thyrocytes could be one main reason why their TSH levels failed to reach the required threshold.

Then how to explain this phenomenon after all? We believed that for one thing, it was possible that thyroid remnant size and/or DTC metastatic lesions could be major factors influencing TSH levels and ablation success rates. For another, several other factors could also influence TSH. For instance, different durations of LT4 withdrawal could affect TSH level. It could be difficult to assure that all patients had the same duration of LT4 withdrawal, since not all of them were very compliant. It is also possible that older patients tend to have a lower ability to produce TSH. In fact, TSH response was found to diminish with age in DTC patients under LT4 therapy^31^. In our study, we found pre-ablation TSH had a threshold value of 99.7 μIU/mL to predict successful ablation with a sensitivity of 57.63% and a specificity of 57.46%. This diagnostic capability was not sufficient, and there was a large overlap in pre-ablation TSH in different groups of patients. Therefore, based on the above evidence, we believe TSH should be considered a second line indicative value and it should not be classified as a strong predictor for successful ablation.

This study has several limitations. First, our study was retrospective in nature. Second, we had only a small sample of 315 patients who fulfilled our inclusion criteria. Third, factors such as distant metastasis and FTC were not analyzed in detail due to small sample scale. Further large-scale prospective studies are needed.

## Conclusion

This study revealed that LN metastasis, pre-ablation Tg and TSH were the most powerful predictors for achieving a disease-free status by the first ^131^I therapy. Patients with LN metastasis, high pre-ablation Tg level and low pre-ablation TSH would be unlikely to achieve a disease-free status.

## Materials and Methods

### Patients

We retrospectively reviewed the DTC patients’ database from the year 2010 to 2015 archive of the Department of Nuclear Medicine, Tianjin Medical University General Hospital. All included patients received total thyroidectomy by our specialized thyroid surgeons. Central neck lymph node removal was conducted in all DTC patients. Unilateral or bilateral neck lymph node dissection of the lateral cervical compartments was performed if the following two conditions were met: 1) clinically or sonographically suspicious lateral lymph nodes were known, 2) pre-operative biopsy or intra-operative excision showed lymph node metastases.

After thyroidectomy, the patients came to our department for ^131^I treatment. The included patients had the first ^131^I therapeutic dose between 100 to 120 mCi. Our exclusion criteria were: 1) less than total thyroidectomy, including subtotal thyroidectomy and lobectomy; 2) thyroglobulin antibody (TgAb) >40 IU/mL prior to the first ^131^I therapy and post-treatment ^131^I whole-body scan, avoiding TgAb’s influence on the veracity of Tg^32^; 3) diagnostic ^131^I whole-body scan was performed before the first ^131^I therapy, ruling out possible stunning effect; 4) patients without adequate data for analysis.

The Institutional Review Board of Tianjin Medical University General Hospital approved the ethical, methodological and protocol aspects of this investigation. All DTC patients provided their written informed consents. We confirm that all methods were carried out in accordance with the relevant guidelines and regulations.

### Classification

For the purpose of our study, we defined risk constellations based on the ATA risk classification^3^,^5^. Specifically, patients with large primary tumor (>4 cm) or extensive extrathyroidal invasion (T4a or T4b in accordance with the TNM system) or with distant metastases were classified as high risk. Patients with non-metastasized intrathyroidal tumors not exceeding a diameter of 4 cm and with <5 central compartment LN metastases and limited extrathyroidal invasion were classified as low risk.

### Protocol

All patients were treated with ^131^I after a preparation of LT4 withdrawal. The patients were asked to keep a strict low iodine diet, and stop using any drug (such as amiodarone) or contrast with iodine. Routine examinations, including serum TSH, Tg, and TgAb levels and cervical ultrasound, were performed. Most of the patients received 100 mCi ^131^I, only 5 patients received 120 mCi due to imaging confirmed existence of metastasis before ^131^I treatment. WBS was performed 4 to 6 days later. In the follow-up treatment, all patients continued TSH suppression therapy, and regular monitoring was conducted in clinic. Further assessments of TSH, Tg, TgAb, cervical ultrasound, and post-treatment whole body scan or diagnostic whole body scan were conducted after 6 to 7 months.

### Serum parameter evaluation

By chemiluminescent reaction principle, free triiodothyronine (reference 3.50–6.50 pmol/L), free thyroxine (reference 11.50–23.50 pmol/L) and TSH (reference 0.30–5.00 μIU/mL, maximum 150.00 μIU/mL) assays were performed on a fully automated ADVIA Centaur analyzer (Siemens Healthcare Diagnostics, New York, USA). Tg (reference 0–55.00 ng/mL, maximum 300.00 ng/mL) and TgAb (reference 0–40.00 IU/mL, maximum 3000.00 IU/mL) were also assessed by chemiluminescent reaction on a fully automated IMMULITE 2000 analyzer (Siemens Healthcare Diagnostics, Los Angeles, USA).

### Predictors and definition

We assessed the following variables as possible influential factors for ^131^I ablation: age, gender, primary tumor size, pathological type, capsular invasion, pathological tumor stage, LN metastasis, distant metastasis, TNM stage, ATA risks, time interval between surgery and ^131^I therapy, pre-ablation Tg, and pre-ablation TSH.

Disease-free status was defined as stimulated Tg < 1 ng/mL, negative TgAb, and no evidence of tumor on cervical ultrasound and WBS 6 to 7 months after first ^131^I therapy^5^.

### Date analysis

Analysis was performed using Statistic Package for Social Science (SPSS) version 17.0. Comparative analyses were performed using independent sample’s t test or chi-square tests. Cutoff values of pre-ablation serum Tg and TSH for diagnosis were determined using ROC analysis. OR with 95% confidence interval was calculated by adopting binary logistic regression. Logistic regression (Forward Wald method) was performed to generate an equation to check parameters’ power for predicting a successful disease-free status. A P value of <0.05 was considered to be significant.

## Additional Information

**How to cite this article**: Liu, N. *et al*. Multiple-factor analysis of the first radioactive iodine therapy in post-operative patients with differentiated thyroid cancer for achieving a disease-free status. *Sci. Rep*. **6**, 34915; doi: 10.1038/srep34915 (2016).

## Figures and Tables

**Table 1:** Comparisons of characteristics of the patients.

Factors	Unsuccessful outcome [Case number (%)]	Successful outcome [Case number (%)]	Statistics	P values
Age				
<45	80(40.61%)	39(33.05%)	X^2^ = 1.794	0.180
≥45	117(59.39%)	79(66.95%)		
Gender				
Male	53(26.90%)	23(19.49%)	X^2^ = 2.215	0.137
Female	144(73.10%)	95(80.51%)		
Size of tumor				
≤1cm	39(19.80%)	36(30.51%)	X^2^ = 4.668	0.031
>1 cm	158(80.20%)	82(69.49%)		
Pathology				
Papillary thyroid cancer	192(97.46%)	118(100.00%)	Fisher exact test	0.161
Follicular thyroid cancer	5(2.54%)	0(0.00%)		
Capsular invasion				
No	113(57.36%)	79(66.95%)	X^2^ = 2.851	0.091
Yes	84(42.64%)	39(33.05%)		
Pathological tumor stage				
T1	65(32.99%)	59(50.00%)	X^2^ = 13.193	0.004
T2	32(16.24%)	19(16.10%)		
T3	87(44.16%)	39(33.05%)		
T4	13(6.60%)	1(0.85%)		
LN* metastasis				
N0	70(35.53%)	62(52.54%)	X^2^ = 18.192	<0.001
N1a	73(37.06%)	46(38.98%)		
N1b	54(27.41%)	10(8.47%)		
Distant metastasis				
M0	183(92.89%)	118(100.00%)	Fisher exact test	0.001
M1	14(7.11%)	0(0.00%)		
TNM* stage				
Stage I	100(50.76%)	63(53.39%)	X^2^ = 4.468	0.215
Stage II	12(6.09%)	8(6.78%)		
Stage III	50(25.38%)	36(30.51%)		
Stage IV	35(17.77%)	11(9.32%)		
ATA risks*				
Low risk	57(28.93%)	55(46.61%)	X^2^ = 17.453	<0.001
Intermediate risk	119(60.41%)	62(52.54%)		
High risk	21(10.66%)	1(0.85%)		
Time interval of surgery and ^131^I therapy				
<3 months	165(83.76%)	100(84.75%)	X^2^ = 0.054	0.816
≥3 months	32(16.24%)	18(15.25%)		
Pre-ablation Tg* (ng/ml)	38.40 ± 72.91	2.50 ± 3.29	t’ = 6.900	<0.001
Pre-ablation TSH* (uIU/ml)	91.98 ± 42.89	107.18 ± 36.11	t’ = −3.367	0.001

*LN = lymph node, TNM stage = American Joint Committee on Cancer tumor-node-metastasis staging system, ATA risks = American Thyroid Association risks, Tg = thyroglobulin, TSH = thyroid stimulating hormone.

**Table 2:** Diagnostic and predictive values of pre-ablation Tg* and TSH* for a successful therapeutic outcome.

	Cutoff values	Accuracy	Sensitivity	Specificity	PPV*	NPV*	AUC* (95% CI*)	P values
Pre-ablation Tg*	3.525 ng/mL	75.24%	77.12%	74.12%	64.09%	84.39%	0.843 (0.801–0.885)	<0.001
Pre-ablation TSH*	99.700 μIU/ml	57.46%	57.63%	57.46%	44.74%	69.33%	0.605 (0.542–0.667)	0.002

*Tg = thyroglobulin, TSH = thyroid stimulating hormone, PPV = positive predictive value, NPV = negative predictive value, AUC = area under the curve, CI = confidence interval.

**Table 3:** Risk assessments on various factors for therapeutic failure.

Factors	OR* (95% CI*)	P values
Age		
<45	1	
≥45	0.722(0.448–1.164)	0.180
Gender		
Female	1	
Male	1.520(0.874–2.645)	0.138
Size of tumor		
≤1 cm	1	
>1 cm	1.779(1.051–3.009)	0.032
Capsular invasion		
No	1	
Yes	1.506(0.935–2.424)	0.092
Pathological tumor stage		
T1	1	
T2	1.529(0.784–2.982)	0.213
T3	2.025(1.208–3.394)	0.007
T4	11.800(1.498–92.978)	0.019
LN* metastasis		
N0	1	
N1a	1.406(0.850–2.324)	0.185
N1b	4.783(2.245–10.190)	<0.001
TNM* stage		
Stage I	1	
Stage II	0.945(0.366–2.440)	0.907
Stage III	0.875(0.514–1.489)	0.623
Stage IV	2.005(0.949–4.232)	0.068
ATA risks*		
Low risk	1	
Intermediate risk	1.852(1.145–2.996)	0.012
High risk	20.263(2.263–155.837)	0.004
Time interval between surgery and ^131^I therapy		
<3 months	1	
≥3 months	1.077(0.575–2.020)	0.816
Pre-ablation Tg* (ng/ml)		
<3.525	1	
≥3.525	9.649(5.651–16.473)	<0.001
Pre-ablation TSH* (uIU/ml)		
<99.700	1	
≥99.700	0.547(0.345–0.867)	0.002

*OR = odds ratio, CI = confidence interval, LN = lymph node, TNM stage = American Joint Committee on Cancer tumor-node-metastasis staging system, ATA risks = American Thyroid Association risks, Tg = thyroglobulin, TSH = thyroid stimulating hormone.
